# Unraveling Daily Linkages between Parenting Behaviors and Adolescent Internalizing Symptoms in Chinese Families: The Mediating Role of Adolescent Self-Compassion

**DOI:** 10.1007/s10964-025-02300-x

**Published:** 2025-12-06

**Authors:** Han Chen, Kaiwen Bi, Paul S. F. Yip, Eddie S. K. Chong

**Affiliations:** Department of Social Work and Social Administration, The University of Hong Kong, Pokfulam, Hong Kong, China

**Keywords:** Parenting, Self-compassion, Internalizing symptoms, Adolescent, Daily diary study, Dynamic structural equation modeling

## Abstract

Parenting behaviors fluctuate within families in daily life, contributing to variations in adolescent psychological adjustment outcomes. While adolescent self-compassion may help explain this link, emerging studies have primarily focused on stable differences between families and examined self-compassion as a mediator at the between-family level. How these mediating processes operate within families, however, remains unknown. To address this gap, this 25-day daily diary study examined within-family level mediation effects of self-compassion on the links between supportive and negative controlling parenting behaviors and adolescent internalizing symptoms. The sample included adolescents (*Mage* = 10.85, *SD* = 0.54; 48.59% boys) and their parents from 155 Chinese families. Positive (self-warmth) and negative (self-coldness) facets of self-compassion were examined as separate constructs. Concurrent mediation results (same-day) generally revealed that adolescents endorsed greater self-warmth on days when they perceived more supportive parenting behaviors, which in turn predicted lower internalizing symptoms. Daily negative controlling parenting behaviors were associated to heightened internalizing symptoms through higher self-coldness on a given day. Lagged indirect effects (next-day) yielded different findings: daily adolescent-perceived supportive parenting behaviors prospectively predicted higher internalizing symptoms two days later via higher next-day self-coldness. Findings highlight the dynamic parenting processes and demonstrate that daily changes in parenting behaviors contribute to adolescent internalizing symptoms via daily shifts in self-compassion.

## Introduction

Internalizing disorders (typically include depression and anxiety) are the most prevalent mental health disorders in adolescents, which can cause functional impairments and increased risk of severe mental health issues in adulthood (Polanczyk et al., 2015). Parenting plays a significant role in influencing adolescent internalizing symptoms (Manuele et al., 2023; Pinquart, 2017). Although attachment theory and empirical evidence suggest that adolescent self-compassion (i.e., a compassionate way of relating to oneself; Neff, 2003) serves as an underlying mechanism for explaining parenting effects on adolescent internalizing symptoms (Bowlby, 1973; Marsh et al., 2018), most studies have treated these variables as stable traits and examined the mediating role of self-compassion at the between-family level (see review: Chen et al., 2025). This not only overlooks the dynamic, day-to-day fluctuations in parenting behaviors and adolescent outcomes within families, but also leaves the mediation role of daily self-compassion unknown. To address this critical gap, the present daily diary study tested within-family level mediating effects of adolescent daily self-compassion in the relationship between daily parenting behaviors and adolescent internalizing symptoms in Chinese families.

### Within-Family Effects of Parenting on Adolescent Internalizing Symptoms

Although previous studies and meta-analyses have recurrently revealed the significant contribution of parenting to adolescent internalizing symptoms (Manuele et al., 2023; Pinquart, 2017), they have primarily viewed parenting behaviors and adolescent psychological outcomes as stable, trait-like properties and focused on how families differ from each other. For instance, in families with greater parental warmth and support and less negative parenting and harsh control, adolescents generally have lower internalizing symptoms than those in other families (Manuele et al., 2023; Pinquart, 2017).

Beyond between-family comparisons, the dynamic systems perspective on adolescent development demonstrates that parenting behaviors and adolescent outcomes are constantly changing as they occur in natural settings (Granic, 2005), necessitating the investigation of parenting effects within the same families. This can help determine, for example, whether adolescents report fewer internalizing symptoms when their own parents are more supportive than usual. This dynamic systems perspective and state-of-the-art analytic approaches have led to a growing body of research examining parenting effects on adolescent development within families. For instance, a systematic review (Boele et al., 2020) synthesizing within-family associations has revealed that more supportive parenting behaviors and fewer negative parent-child interactions are associated with decreased adolescent internalizing problems at the within-family level. However, as most studies examining within-family level effects of parenting have been conducted in Western countries (Boele et al., 2020), it is essential to explore these within-family relationships and the underlying mechanism in culturally distinct contexts such as China. This is important because the same parenting behaviors may have distinct meanings and effects on adolescents in different cultural contexts (Chao, 1994).

Considering that everyday influences and interactions between parents and their adolescents are “the primary engines of development” (Bronfenbrenner, 2005, p. 6), examining parenting effects at the daily level is crucial. A daily diary approach is suited, which is well positioned to zoom into micro, real-time processes in everyday family lives. The daily diary method can also enhance ecological validity and mitigate the influence of retrospective memory bias (Beals et al., 2009).

### Daily Self-Compassion as a Within-Family Mediator

Although an increasing number of studies has revealed significant within-family level relationship between parenting and adolescent psychological adjustment (Boele et al., 2020; Boele et al., 2023a), the underlying mechanism has remained unclear. Attachment theory and empirical evidence suggest that adolescent self-compassion might be a mediating mechanism through which parenting exerts effects. Self-compassion refers to how individuals relate to themselves and is operationalized as a multifaceted construct comprising three domains with a positive and a negative facet each (Neff, 2003): (1) whether individuals kindly respond to their own suffering (self-kindness vs self-judgment), (2) whether people cognitively recognize life challenges as shared human experience (common humanity vs isolation) and (3) whether individuals could be open and present with their pain (mindfulness vs overidentification). Although self-compassion is typically indicated by the average score of all six components (Neff, 2003), self-warmth (i.e., self-kindness, common humanity and mindfulness) and self-coldness (i.e., self-judgment, isolation and overidentification) may involve two distinct affect systems. Self-warmth relates to the safeness-soothing system which functions to promote self-soothing, calming and contentment, while self-coldness is associated with the threat system, which has evolved to be attuned to potential threats (Gilbert, 2009). Empirical studies have also demonstrated that self-warmth and self-coldness are two distinct constructs by showing a better goodness-of-fit for a two-factor structure (i.e., self-warmth and self-coldness) than a one-factor structure (Brenner et al., 2017) and their differential predictability of mental outcomes (Chio et al., 2021). Treating self-warmth and self-coldness as separate constructs might be particularly important in Eastern societies such as China because people from this dialectical culture tend to adopt a dialectical thinking style, enabling them to endorse both positive and negative dimensions of self-compassion simultaneously instead of merely maximizing one facet while minimizing the other (Chio et al., 2021; Wu et al., 2020).

The beneficial psychological effects of self-compassion have been well documented across different populations, including adolescents (Marsh et al., 2018). Attachment theory (Bowlby, 1973) provides a theoretical framework for further explaining how parenting contributes to adolescent psychological outcomes via adolescent self-compassion. According to the attachment theory, parenting behaviors may play a significant role in affecting adolescent self-compassion through influencing adolescent internal working model, referring to mental representations that provide cognitive and affective templates for relating to oneself and others (Bowlby, 1973). Supportive parenting behaviors, such as parental warmth (i.e., parental affection, care and responsiveness) and autonomy support (i.e., encouragement of independent decision-making, acknowledgment and interest in adolescents’ perspectives) (Soenens et al., 2017), give rise to positive internal working models of adolescents themselves as valued and worthy of care (Raque et al., 2023) and foster a self-caregiving mental representation (Mikulincer & Shaver, 2004). These enable adolescents to soothe and reassure themselves when facing distress. Conversely, adolescents exposed to parental negative controlling behaviors such as psychological control (i.e., parents manipulating adolescents via verbal expression constraint, guilt induction and love withdrawal; Barber, 1996) and strictness (i.e., parents setting stringent requirements and imposing their expectations on their adolescent by asserting their authority; Garcia et al., 2024), are prone to form negative internal working models of themselves as incompetent and unworthy of having their needs met. This negative self-representation impairs their ability to recognize their need for care and to generate compassion for themselves, instead leading to an uncompassionate and critical self-to-self relating internalized from parental negative treatment (Mikulincer & Shaver, 2004; Song et al., 2023). However, since internal working models are primarily assumed to be stable, global traits formed through early caregiving experiences (Mikulincer & Shaver, 2007), an unknown yet vital question is whether parenting behaviors can have short-term effects on adolescent internal working models and, consequently, their self-compassion in daily life.

Emerging studies have provided empirical support for the significant mediation role of self-compassion in the relationship between parenting behaviors and adolescent internalizing symptoms (Chen et al., 2025). However, they have primarily focused on stable differences between families. Considering the valence of between-family associations may be different, or even be inverted, at the within-family level (e.g., Keijsers, 2016), it is essential to disentangle within-family from between-family effects and explore how the mediation role of self-compassion operates within families in daily life. The predominant use of cross-sectional design in past studies also precludes the possibility to clarify the temporal sequences among variables (see review: Chen et al., 2025). Employing a daily diary study design can address this limitation by examining time-lagged mediating effects from one day to the next. However, since parenting is a dynamic process and its effects can vary differently over time in daily life (Granic, 2005), the associations between parenting behaviors and adolescent outcomes can differ in both significance and valence (i.e., positive or negative) at varying time intervals (Boele et al., 2023b). To more precisely capture the dynamic daily parenting processes, both concurrent (same-day) and lagged (next-day) mediating effects need to be examined to investigate how parenting behaviors co-occurred with and prospectively predicted adolescent internalizing symptoms through adolescent self-compassion. Prior research on the mediation role of self-compassion has mostly combined maternal and paternal parenting or focused solely on maternal parenting (Temel & Atalay, 2020; Neff & McGehee, 2010; Zhao et al., 2023), limiting the understanding of how paternal parenting affects adolescent self-compassion and internalizing outcomes. This suggests that more research attention should be paid to fathers’ parenting behaviors.

Empirical experience sampling studies have supported the dynamic system perspective by revealing within-family level variations in parenting, self-compassion and psychological outcomes (Boele et al., 2020; Mey et al., 2023) and provided some evidence for within-family level mediation effect of self-compassion. For instance, an ecological momentary assessment study has shown the positive effect of state self-compassion on momentary affective well-being (Mey et al., 2023). A daily diary study has found that higher daily self-compassion predicts decreased perceived daily stress (Li et al., 2020). Despite substantial empirical support for within-family association between self-compassion and psychological outcomes, there is a dearth of research investigating the association between parenting and self-compassion within families. One exception is a 4-year longitudinal study (Kaufmann et al., 2023), which found that in years when adolescents perceived higher parental support than usual, they exhibited greater self-warmth and lower self-coldness. The opposite pattern was identified for parental psychological control. Although this study distinguished the within- and between-effects of parenting on adolescent self-compassion, it employed a macro timescale (i.e., yearly). However, family processes detected at the macro timescales may not necessarily apply to the micro timescale (e.g., seconds, hours, days; Boele et al., 2023b), resulting in the short-term effects of daily parenting behaviors on adolescent self-compassion, as well as the mediating effect of daily self-compassion, unknown.

## Current Study

From a dynamic systems perspective, emerging studies have shown significant within-family association between parenting and adolescent psychological adjustment. However, the underlying mechanisms have been underexplored. Guided by the attachment theory, this daily diary study addressed this gap by examining the within-family mediation effect of adolescent self-compassion in relationships between parenting behaviors and internalizing symptoms in daily life. Considering potential differences in parenting effects on adolescent outcomes at different time intervals, both same-day and next-day mediation effects were tested. Two facets of self-compassion (i.e., self-warmth and self-coldness) were analyzed as distinct constructs. Considering the inadequate attention paid to effects of paternal parenting, both mothers’ and fathers’ parenting behaviors were assessed. Based on attachment theory and existing evidence, the following hypotheses were made. For same-day mediation effects, two supportive parenting behaviors (i.e., warmth and autonomy support) would be associated with higher same-day adolescent self-warmth, which in turn would contribute to lower levels of internalizing symptoms on that day (Hypothesis 1). Conversely, two negative and controlling parenting behaviors (i.e., psychological control and strictness) would predict higher self-coldness on a given day, which were then linked to increased same-day internalizing symptoms (Hypothesis 2). For the next-day mediation effects, no specific hypotheses were made due to limited evidence.

## Methods

### Participants and Procedure

Families were recruited from an elementary school in the urban area of Southwestern China. Families were invited to complete daily diaries across 21 consecutive days. During the diary completion period, participants received an initial prompt at around 5 pm each day and were reminded twice thereafter. Participants were permitted to complete the daily survey until 3:00 am, after which the submission link expired. Dairies submitted between 00:00 am and 3:00 am were treated as diaries on the previous day. If the same individual submitted multiple diaries on a given day, only the first submission was retained while the other submission(s) on the same day were deleted. To improve statistical power and increase the total sample size (i.e., N × number of completed diaries per N), participants were encouraged to continue submitting diaries after the initial 21 days, with a maximum of 25 days allowed. Ethical approval for the present study was obtained from the Institutional Review Board of the University of Hong Kong.

One adolescent from a pair of siblings in the dataset was randomly selected and removed using R programming (set.seed = 20240613). A total of 155 families were included as the final analytic sample. Adolescents from these families had a mean age of 10.85 years (*SD* = 0.54) and 48.59% were boys. Socio-demographic information of parents is presented in Supplementary Table S1.

### Measures

#### Daily parenting behaviors

Daily parenting scales utilized in a previous daily diary study (Boele et al., 2023a) were translated into Chinese and used for assessing four daily parenting behaviors. Average scores of the relevant items were utilized to represent the corresponding parenting behaviors. Participants rated these items on a Likert scale ranging from 1 (Not at all) to 10 (Very much).

##### Parental warmth

Adolescent-perceived daily maternal or paternal warmth were measured by two items. Sample item was “Today, my mother/father showed me that she/he cared for me”. Items were adapted to assess parent-reported parenting behaviors from parents’ own perspectives. The reliabilities for maternal warmth were *ω*^W^_adolescent_ = 0.57, ω^B^_adolescent _= 0.85, *ω*^W^_mother _= 0.64, *ω*^B^_mother _= 0.96. The reliabilities for paternal warmth were *ω*^W^_adolescent_ = 0.81, *ω*^B^_adolescent_ = 0.89, *ω*^W^_father _= 0.71, *ω*^B^_father_ = 0.95.

##### Parental autonomy support

This was assessed by two items. For adolescent-perceived parental autonomy support, sample item was “Today, my mother/father allowed me to make my own plans”. The reliabilities for maternal autonomy support were *ω*^W^_adolescent_ = 0.80, *ω*^B^_adolescent _= 0.92, *ω*^W^_mother _= 0.60, *ω*^B^_mother_ = 0.95. The reliabilities for paternal autonomy support were *ω*^W^_adolescent_ = 0.83, *ω*^B^_adolescent _= 0.94, *ω*^W^_father_ = 0.68, *ω*^B^_father_ = 0.95.

##### Parental psychological control

Three items (e.g., “Today, my parent was less affectionate toward me when I did not see things his/her way”) were used to assess daily parental psychological control. The reliabilities for maternal psychological control were *ω*^W^_adolescent _= 0.71, *ω*^B^_adolescent_ = 0.82, *ω*^W^_mother _= 0.53 and *ω*^B^_mother _= 0.86. The reliabilities for paternal psychological control were *ω*^W^_adolescent_ = 0.78, *ω*^B^_adolescent_ = 0.94, *ω*^W^_father_ = 0.58 and *ω*^B^_father_ = 0.90.

##### Parental strictness

This was measured by one item. For example, adolescent-perceived strictness was assessed by “My mother/father was strict”.

#### Daily self-compassion

The six-item State Self-Compassion Scale-Short form (SSCS-S; Neff et al., 2021) was adapted to assess daily self-compassion. Specifically, items of state self-compassion used present moment language (e.g., “I’m giving myself the caring and tenderness I need.”) and these items were adapted to ask about adolescents’ experiences in relating to themselves on a given day (e.g., “Today, I gave myself the caring and tenderness”). Daily self-warmth was assessed by three positive items while daily self-coldness was measured by the other three negative items (e.g., “Today, I felt intolerant and impatient toward myself”). The average scores of corresponding items were used to indicate self-warmth and self-coldness levels. The reliabilities were *ω*^W^_adolescent _= 0.64, *ω*^B^_adolescent _= 0.83 for self-warmth, and were *ω*^W^_adolescent _= 0.60, *ω*^B^_adolescent _= 0.82 for self-coldness. Adolescents reported their responses on a Likert scale from 1 (not at all true for me) to 5 (very true for me).

#### Daily internalizing symptoms

The Patient Health Questionnaire-4 (PHQ-4; Kroenke et al., 2009) was used to assess daily internalizing symptoms. Adolescents rated these 4 items on a 10-point Likert scale from 1 (not at all) to 10 (very much). The average scores of two items were used to indicate depression symptoms (e.g., “Today, I felt down, depressed, or hopeless”). The mean scores of the other two items were used to represent anxiety symptoms (e.g., “Today, I felt nervous, anxious or on edge”). The reliabilities were ω^W^_adolescent_ = 0.85, ω^B^_adolescent _= 0.96 for depression symptoms, and were ω^W^_adolescent_ = 0.83, ω^B^_adolescent _= 0.96 for anxiety symptoms.

### Statistical Analysis

Considering the multilevel structure of data and the attempt to test both concurrent and lagged mediation effects, Dynamic Structural Equation Modeling (DSEM) approach was employed because it combines multilevel modeling (enabling the disentanglement of between- and within-family effects), time-series analysis (allowing the modeling of lagged associations) and structural equation modeling (Asparouhov et al., 2018). Analyses were conducted using M*plus* 8.3 with Bayesian estimation.

All hypotheses were initially examined using adolescents’ reports. Considering the high correlations between maternal and paternal parenting behaviors, they were examined in separate models to avoid multicollinearity. Each maternal or paternal parenting behavior (i.e., warmth, autonomy support, psychological control and strictness) was examined in separate models. Self-warmth and self-coldness were examined as two mediators in the same model. Depression symptom was tested as internalizing symptom in each model. Two sets of mediation models with different time intervals were examined: (1) concurrent mediation model (i.e., testing associations among all variables and the mediating effects at the same day t) (2) lagged mediation model (testing the mediating effects of self-warmth and self-coldness at day t - 1 in the relationship between parenting behaviors at day t - 2 and adolescent depression symptom at day t). Both concurrent and lagged mediation model included autoregressive paths from each variable (parenting behaviors, self-warmth, self-coldness and depression symptom) at day t -1 to itself at day t.

Since the average within-family effects were the main focus of this study, the DSEM mediation models included random intercepts (i.e., the between-family variances in the outcomes separated from the within-family variances) and fixed slopes (i.e., within-family direct effects were assumed to be fixed and not varying between families), which can help prevent unnecessary complications and improve model convergence (Preacher et al., 2010). Each model was run with 10,000 iterations, 2 thinning factors and 2 chains. The convergence was checked based on three criteria: (1) Gelman–Rubin Potential Scale Reduction Factor (PSRF) values close to 1; (2) trace plots (i.e., no upward or downward trend of parameters with two chains overlapping well) and (3) autocorrelation plots with values around or below 0.1. All DSEM models achieved excellent convergence across all criteria. Effects were deemed as significant if the 95% credible intervals (CI) did not include zero.

DSEM is well equipped to deal with missing data using Bayesian methods. The missingness was handled by the Markov Chain Monte Carlo (MCMC) method under Bayesian estimation (Asparouhov & Muthén, 2021). Specifically, model was estimated based on MCMC algorithm that arranged all model parameters and missing data into blocks. A new value for block elements, including missing data, was constantly generated, which was conditional on all the other blocks and the data. This process was repeated until a stable posterior distribution was obtained (Asparouhov et al., 2018). To address unequal time intervals between observations due to missing data, the TINTERVAL was set to 1 (representing one day). This ensured that all data were placed in the equal time interval of one day and missing data were inserted into days without data (Hamaker et al., 2018).

### Exploratory Analysis

Two sets of exploratory analyses were included: (1) To explore the robustness of findings across different internalizing symptoms, adolescent anxiety symptom was also tested as the outcome in addition to depression symptoms examined in the main analysis. (2) Since past studies on the mediating effects of self-compassion have solely relied on adolescents’ reports of parenting (Temel & Atalay, 2020; Zhao et al., 2023), the present study addressed this limitation by employing a multi-informant approach and assessing parenting behaviors from both adolescents’ and parents’ reports. This is important because parents and adolescents frequently present discordant perceptions of parenting behaviors (Xu & Zheng, 2022).

## Results

### Compliance and Missing Data

Among 155 families included in this study, 144 adolescents participated in this 25-day daily diary study and completed 15.89 diaries on average (*SD* = 8.14, range = 1 to 25), with a completion rate of 63.56%. Of the surveys completed by adolescents, 11.66% missingness was found across all variables (maternal and paternal parenting behaviors, self-warmth, self-coldness, depression and anxiety symptoms) over 25 days. A total of 129 mothers completed 16.70 diaries on average (*SD* = 8.11, range = 1 to 25) with no missing data in completed surveys (completion rate = 66.80%). 113 fathers participated and completed an average of 15.30 diaries (*SD* = 8.59, range = 1 to 25) with no missing data in completed surveys (completion rate = 61.20%). The completion rates were acceptable and comparable to those in previous experience sampling studies (Van der Kaap-Deeder et al., 2023).

### Preliminary Results

Table S2 in the supplementary material presents descriptives, bivariate correlations and intraclass correlation coefficients (ICCs) among main variables. ICC indicates the proportion of variance explained by between-family differences. Correspondingly, the value (1 - ICC) represents the proportion of within-family variances plus error. According to ICCs, 30% to 53% variances in adolescent-perceived parenting behaviors and 27% to 40% variances in parent-reported parenting behaviors could be attributed to within-family fluctuations. Substantial within-family level variances were detected for self-warmth (40%) and self-coldness (41%). Nearly half to over half of the variances in adolescent depression (50%) and anxiety symptoms (52%) were due to within-family level variations.

### DSEM Results Based on Adolescent-Reported Parenting

#### Concurrent mediation effects

As shown in Table 1, Adolescents who perceived higher maternal warmth on a given day had higher than usual levels of self-warmth, which in turn was linked to lower depression symptoms on that day. Indirect effect showed that self-warmth significantly mediated the concurrent relationship between maternal warmth and depression symptoms at the within-family level. Adolescent self-warmth was higher than usual on days when they perceived higher maternal autonomy support. Adolescents also endorsed higher daily self-coldness on days characterized by higher perceived maternal autonomy support despite the smaller effect size compared with self-warmth. Adolescents reported less depression symptoms on days when they had higher self-warmth and lower self-coldness. Hence, both self-warmth and self-coldness were significant mediators in the contemporaneous link between maternal autonomy support and adolescent depression symptoms at the within-family level. Adolescents who perceived higher daily maternal psychological control reported higher daily self-coldness and self-warmth, although the latter was unexpected. Self-coldness and self-warmth were associated with higher and lower same-day depression symptoms, respectively. As such, both facets of self-compassion significantly mediated the within-family level relationship between maternal psychological control and adolescent depression symptoms on the same day. On days when adolescents perceived higher strictness from their mothers, they reported higher self-coldness, which was in turn linked to greater depression symptoms on that day. Indirect effect indicated that daily self-coldness significantly mediated the concurrent association between maternal strictness and adolescent depression symptoms.

**Table 1:** Concurrent Within-Family Level Mediation Results for Adolescent-Reported Parenting with Adolescent Depression Symptom as the Outcome

	Path a1 (parenting → SW)		Path a2 (parenting → SC)		Path b1 (SW → Dep)		Path b2 (SC → Dep)		Path c’ (parenting → Dep)		Indirect effect 1 (mediator: SW)		Indirect effect 2 (mediator: SC)	
Model	β	95% CI	β	95% CI	β	95% CI	β	95% CI	β	95% CI	*B*	95% CI	*B*	95% CI
Maternal parenting														
Warmth → SW and SC → Dep	**0.14**	**[0.09, 0.18]**	0.04	[0.00, 0.08]	−**0.10**	**[**−**0.14**, −**0.06]**	**0.28**	**[0.25, 0.32]**	−0.01	[−0.05, 0.03]	−**0.01**	**[**−**0.02**, −**0.008]**	0.01	[0.00, 0.02]
Autonomy support → SW and SC → Dep	**0.17**	**[0.13, 0.22]**	**0.07**	**[0.02, 0.11]**	−**0.10**	**[**−**0.14**, −**0.06]**	**0.28**	**[0.25, 0.32]**	−0.03	[−0.07, 0.01]	−**0.02**	**[**−**0.03**, −**0.01]**	**0.02**	**[0.01, 0.03]**
Psychological control → SW and SC → Dep	**0.07**	**[0.02, 0.11]**	**0.13**	**[0.09, 0.17]**	−**0.11**	**[**−**0.15**, −**0.08]**	**0.26**	**[0.22, 0.30]**	**0.18**	**[0.14, 0.22]**	−**0.01**	**[**−**0.02**, −**0.004]**	**0.05**	**[0.03, 0.07]**
Strictness → SW and SC → Dep	0.03	[−0.01, 0.08]	**0.09**	**[0.05, 0.13]**	−**0.11**	**[**−**0.15**, −**0.07]**	**0.27**	**[0.24, 0.31]**	**0.10**	**[0.06, 0.14]**	−0.003	[−0.01, 0.001]	**0.02**	**[0.01, 0.04]**
Paternal parenting														
Warmth → SW and SC → Dep	**0.17**	**[0.13, 0.22]**	**0.05**	**[0.003, 0.10]**	−**0.09**	**[**−**0.13**, −**0.05]**	**0.29**	**[0.25, 0.32]**	−**0.07**	**[**−**0.11**, −**0.02]**	−**0.02**	**[**−**0.03**, −**0.01]**	**0.01**	**[0.001, 0.03]**
Autonomy support → SW and SC → Dep	**0.19**	**[0.15, 0.24]**	0.05	[0.00, 0.09]	−**0.09**	**[**−**0.13**, −**0.05]**	**0.28**	**[0.25, 0.32]**	−**0.06**	**[**−**0.10**, −**0.01]**	−**0.02**	**[**−**0.03**, −**0.01]**	0.01	[0.00, 0.03]
Psychological control → SW and SC → Dep	−0.003	[−0.05, 0.04]	**0.16**	**[0.11, 0.21]**	−**0.10**	**[**−**0.14**, −**0.06]**	**0.25**	**[0.22, 0.29]**	**0.17**	**[0.12, 0.21]**	0.00	[−0.01, 0.01]	**0.06**	**[0.04, 0.08]**
Strictness → SW and SC → Dep	0.01	[−0.03, 0.06]	**0.09**	**[0.05, 0.14]**	−**0.10**	**[**−**0.14**, −**0.07]**	**0.27**	**[0.23, 0.31]**	**0.12**	**[0.07, 0.16]**	−0.001	[−0.01, 0.003]	**0.03**	**[0.01, 0.04]**

β for each path was standardized coefficient; *B* for each indirect effect was unstandardized coefficient. The boldface coefficients were significant according to 95% credible intervals

SW = self-warmth; SC = self-coldness; Dep = depression

As for paternal parenting, adolescents who reported higher daily paternal warmth exhibited higher levels of self-warmth on that day. Daily paternal warmth was also significantly associated with higher daily self-coldness. Daily self-warmth and self-coldness in turn contributed to lower and higher levels of same-day depression symptoms, respectively. As a result, both self-warmth and self-coldness significantly mediated the concurrent relationship between paternal warmth and adolescent depression symptoms at the within-family level. Adolescents reported higher self-warmth on days featuring higher levels of paternal autonomy support. Higher daily self-warmth was in turn associated with lower levels of same-day depression symptoms. Indirect effect showed that daily self-warmth significantly mediated the contemporaneous association between paternal autonomy support and depression symptoms. In addition, on days when adolescents perceived their fathers exerted greater psychological control, they reported higher self-coldness, which in turn contributed to higher same-day depression symptoms. Similar patterns were found for perceived paternal strictness. As such, daily self-coldness significantly mediated the concurrent association between paternal psychological control and adolescent depression symptoms, and between paternal strictness and depression symptoms. As shown in Supplementary Table S3, the concurrent mediation results with adolescent anxiety symptom as the outcome were consistent with the above results focusing on depression symptom.

#### Lagged mediation effects

Only three significant indirect effects were found when testing the mediating effects prospectively (see Table 2). Specifically, adolescent-perceived maternal autonomy support at day t-2 was significantly associated to higher levels of adolescent self-coldness at day t-1, which in turn predicted higher levels of depression symptoms at day t. Similar effects were observed for adolescent-reported paternal warmth and paternal autonomy support. Therefore, daily self-coldness at day t-1 significantly mediated the lagged associations between adolescent-perceived maternal autonomy support, paternal warmth and autonomy support and adolescent depression symptoms. Results were comparable when testing adolescent anxiety symptoms as the outcome (see Supplementary Table S4).

**Table 2:** Lagged Within-Family Level Mediation Results for Adolescent-Reported Parenting with Adolescent Depression Symptom as the Outcome

	Path a1 (parenting → SW)		Path a2 (parenting → SC)		Path b1 (SW → Dep)		Path b2 (SC → Dep)		Path c’ (parenting → Dep)		Indirect effect 1 (mediator: SW)		Indirect effect 2 (mediator: SC)	
Model	*β*	95% CI	*β*	95% CI	*β*	95% CI	*β*	95% CI	*β*	95% CI	*B*	95% CI	*B*	95% CI
Maternal parenting														
Warmth (t-2) → SW and SC (t-1) → Dep (t)	0.03	[−0.01, 0.08]	0.04	[−0.01, 0.08]	0.02	[−0.03, 0.06]	**0.07**	**[0.02, 0.11]**	0.04	[−0.01, 0.08]	0.00	[−0.001, 0.003]	0.002	[0.00, 0.01]
Autonomy support (t-2) → SW and SC (t-1) → Dep (t)	0.04	[−0.01, 0.09]	**0.06**	**[0.02, 0.11]**	0.02	[−0.02, 0.07]	**0.07**	**[0.02, 0.12]**	−0.02	[−0.07, 0.02]	0.001	[−0.001, 0.004]	**0.004**	**[0.001, 0.01]**
Psychological control (t-2) → SW and SC (t-1) → Dep (t)	0.02	[−0.03, 0.07]	0.01	[−0.03, 0.06]	0.02	[−0.03, 0.06]	**0.07**	**[0.02, 0.12]**	−0.02	[−0.07, 0.02]	0.00	[−0.001, 0.004]	0.001	[−0.004, 0.01]
Strictness (t-2) → SW and SC (t-1) → Dep (t)	0.03	[−0.02, 0.07]	−0.01	[−0.05, 0.04]	0.01	[−0.03, 0.06]	**0.07**	**[0.02, 0.12]**	−0.004	[−0.05, 0.04]	0.00	[−0.001, 0.002]	0.00	[−0.004, 0.003]
Paternal parenting														
Warmth (t-2) → SW and SC (t-1) → Dep (t)	0.05	[0.00, 0.10]	**0.07**	**[0.03, 0.12]**	0.02	[−0.02, 0.07]	**0.06**	**[0.02, 0.11]**	0.03	[−0.02, 0.08]	0.001	[−0.001, 0.01]	**0.004**	**[0.001, 0.01]**
Autonomy support (t-2) → SW and SC (t-1) → Dep (t)	0.05	[0.00, 0.10]	**0.07**	**[0.02, 0.12]**	0.02	[−0.02, 0.07]	**0.06**	**[0.02, 0.11]**	0.04	[−0.02, 0.09]	0.001	[−0.001, 0.01]	**0.004**	**[0.001, 0.01]**
Psychological control (t-2) → SW and SC (t-1) → Dep (t)	0.01	[−0.04, 0.06]	0.03	[−0.02, 0.08]	0.02	[−0.03, 0.06]	**0.07**	**[0.02, 0.11]**	0.03	[−0.02, 0.08]	0.00	[−0.002, 0.003]	0.003	[−0.002, 0.01]
Strictness (t-2) → SW and SC (t-1) → Dep (t)	−0.002	[−0.05, 0.05]	0.02	[−0.03, 0.07]	0.02	[−0.03, 0.06]	**0.07**	**[0.03, 0.12]**	−0.02	[−0.07, 0.03]	0.00	[−0.002, 0.002]	0.001	[−0.002, 0.01]

β for each path was standardized coefficient; *B* for each indirect effect was unstandardized coefficient. The boldface coefficients were significant according to 95% credible intervals

SW = self-warmth; SC = self-coldness; Dep = depression

### DSEM Results Based on Parent-Reported Parenting (Exploratory)

Only one significant concurrent mediation effect was found for parent-reported parenting at the within-family level: higher paternal strictness on a certain day significantly predicted higher levels of same-day adolescent self-coldness, which in turn contributed to higher levels of depression and anxiety symptoms on that day (see Supplementary Tables S5, S7). Indirect effect indicated that adolescent daily self-coldness significantly mediated the contemporaneous association between father-reported paternal strictness and adolescent internalizing symptoms. No significant lagged indirect effects were observed based on parent-reported parenting behaviors (see Supplementary Tables S6, S8).

## Discussion

The mediation effect of adolescent self-compassion on the relationship between parenting behaviors and adolescent internalizing symptoms has not been established within families. This daily diary study filled this gap by testing daily self-compassion as the within-family mediator. Both concurrent (same-day) and lagged (next-day) associations among daily parenting behaviors, adolescent self-compassion and internalizing symptoms were examined to investigate whether time interval matters. Concurrent mediating effects were mostly aligned with hypotheses, while different results emerged for the lagged mediation effects. Discussion of results was situated within the broader Chinese cultural context.

### Same-Day Mediation Effects

Same-day mediation effects were mostly aligned with our hypotheses. Based on adolescent-perceived parenting behaviors, consistent patterns were found across maternal and paternal parenting behaviors: on days when adolescents perceived higher-than-usual parental warmth and autonomy support, they were more inclined to exhibit self-warmth, which in turn related to lower levels of internalizing symptoms on that day. Conversely, on days featuring greater perceived parental psychological control or strictness, adolescents endorsed higher self-coldness, which was in turn associated to elevated same-day internalizing symptoms. These findings extend attachment theory by demonstrating that its tenets hold at the short-term, within-family level. Instead of being stable across lifespan and various contexts (Mikulincer & Shaver, 2007), internal working models may vary over time in response to daily parenting behaviors. Daily perceptions of supportive parenting behaviors may have an immediate effect on adolescent internal working model, reinforcing a positive view of themselves as deserving of care and nurturing on that day. The warmth, care and support perceived from parents on a given day can also serve as a compassionate template for adolescents to form a self-caregiving mental representation, enabling them to treat themselves with kindness and respond to their struggles in a compassionate way on that day. This compassionate self-responding can foster adolescents’ resilience when confronted with daily hassles, thereby reducing daily internalizing symptoms (Brenner et al., 2018). Daily negative controlling parenting behaviors may adversely impact adolescents’ internal working models, prompting adolescents to view themselves as incompetent and undeserving of support and affection on that day. This negative internal working model of the self can undermine adolescents’ self-compassion by inhibiting their ability to notice their own distress and acknowledge their needs for care and compassion (Song et al., 2023). The harshness and negative control perceived from daily parental behaviors can be internalized by adolescents, resulting in a similar critical and judgmental way to respond to themselves on that day (Mikulincer & Shaver, 2004). This uncompassionate self-responding can in turn exacerbate same-day internalizing symptoms of adolescents.

Several concurrent mediation effects were opposite to the hypotheses. Specifically, although greater daily adolescent-perceived maternal autonomy support and paternal warmth contributed to lower internalizing symptoms through higher self-warmth as hypothesized, they also predicted higher internalizing symptoms via increased self-coldness on a given day. The unexpected positive link between parental supportive behaviors and self-coldness may be attributed to the positive connotations of self-coldness in Chinese culture. Influenced by Confucianism, emotions reflecting individuals’ flaws or lack of competence are often considered desirable and acceptable (Eid & Diener, 2001). For example, self-criticism and self-judgment are viewed as essential ways for Chinese people to deeply reflect on their past faults or failures, which can prepare them for correcting mistakes and transforming negative experiences to positive ones (Wu et al., 2020). When Chinese adolescents perceive higher warmth and autonomy support from their parents on a certain day, they might feel a greater necessity to uphold high personal standards and facilitate personal performance by fixing attention on their inadequacies and negative experiences–a process motivated by a desire to repay their parents’ affection, care and trust for their independent decisions (Yeh & Bedford, 2004). Another unexpected mediation result is that adolescent-perceived maternal psychological control contributed to decreased same-day internalizing symptoms via higher self-warmth, though the hypothesized mediating path through self-coldness was also significant. The former result might be explained by adolescents’ positive interpretations of parental psychological control as indicators of parental concern, care and involvement in Chinese cultural context (Chao, 1994). These unexpected concurrent results found in Chinese adolescents underscore culture as a prime context for understanding daily family processes, as it shapes meanings of certain parenting behaviors and determines their connections with adolescent daily outcomes.

Most significant within-family level mediation effects were found using adolescent-perceived rather than parent-reported parenting behaviors. This aligns with previous longitudinal study (Van Lissa et al., 2019), collectively indicating adolescent-perceived parenting as the primary source for determining adolescent socioemotional and mental health outcomes. Only one significant mediation effect was observed based on parent-reported parenting: daily paternal strictness was associated with higher same-day adolescent depression and anxiety symptoms via heightened self-coldness. The robustness of this mediation effect across adolescents’ and parents’ reports of parenting highlights that fathers’ assertion of authority and exhibition of strictness in daily life might be particularly influential in fostering negative internal working models of adolescents, thereby elevating adolescents’ self-coldness and internalizing symptoms.

### Time Interval Matters: Different Next-Day Mediation Effects

Dynamic systems theory posits that real-time behaviors are variable and within-family processes unfold differently at varying time intervals (Granic, 2005). The present study supports this claim by identifying differences in valence and statistical significance between concurrent and lagged mediation associations. Specifically, despite few unexpected findings, concurrent results generally support the prevailing belief about the beneficial effects of supportive parenting behaviors and deleterious effects of negative controlling parenting behaviors. However, these parenting effects on adolescent self-compassion and internalizing symptoms may be immediate yet transient, which cannot sustain until the following day. For negative parenting behaviors, this may be attributable to adolescents’ adoption of adaptive coping skills to mitigate the adverse effects on next-day self-compassion. Regarding supportive parenting behaviors, this may be explained by the fact that their positive effects on adolescent self-warmth may be less durable than their predictability of adolescent self-coldness, as the latter exhibits significant persistence into the next day. The significant lagged indirect results show the lasting effects of parental supportive behaviors on adolescent internalizing symptoms through heightened self-coldness. This could be explained by reciprocal filial piety endorsed by Chinese adolescents. After perceiving parents’ warmth, care and encouragement of autonomy, adolescents might transform these supportive parenting behaviors to higher motivations of improving their personal performance and growth the next day (e.g., pursuing better academic performance that is often tied to family honor in Chinese families) as a way of expressing their gratitude and love for their parents’ benefaction (Yeh & Bedford, 2004). However, as illustrated previously, adolescents from Chinese cultures tend to adopt an uncompassionate self-responding as a self-improvement tool, despite its detrimental psychological effects.

### Distinctions between Two Facets of Self-Compassion

Concurrent results reveal more pronounced effects of supportive parenting behaviors on self-warmth and more consistent effects of negative controlling parenting behaviors on self-coldness. Although several results were opposite to hypotheses (e.g., positive association between adolescent-perceived maternal autonomy support and self-coldness), the smaller magnitude of this relationship compared to the corresponding positive association with self-warmth supports this claim. This might be explained by the fact that self-warmth and self-coldness are rooted in two distinct processing systems (i.e., safeness-soothing system and the threat-defense system; Gilbert, 2009). Supportive parenting behaviors foster emotional safeness in adolescents, activating their safeness-soothing system that is responsible for generating self-warmth (Gilbert et al., 2011). Nevertheless, the absence of supportive parenting does not necessarily induce emotional threat leading to self-coldness. Similarly, negative controlling parenting can elicit emotional threat, which triggers threat-defense system for elevating self-coldness. The lack of negative controlling parenting behaviors, however, does not automatically create emotional safeness that fosters self-warmth. According to lagged effects, the long-lasting effects of parenting behaviors are more evident for self-coldness, which may be explained by the positive meanings ascribed to self-coldness in Chinese culture. Aligned with previous findings (Chio et al., 2021), both concurrent and lagged results of the current study indicate that self-coldness has a greater predictability of adolescent internalizing symptoms than self-warmth. The differential linkages between two facets of self-compassion and both parenting behaviors and internalizing symptoms illustrate the necessity of conceptualizing and analyzing self-warmth and self-coldness as distinct constructs.

### Implications

This daily diary study provides vital implications for developmental research and theory. The present study reveals day-to-day variations in parenting behaviors, adolescent self-compassion and internalizing symptoms (as evidenced by ICCs) and daily associations among these variables, providing empirical support for dynamic systems perspective (Granic, 2005). This challenges the predominant approach in developmental field, which conceptualizes parenting and adolescent outcomes as stable trait-like properties and merely focuses on between-family differences. Developmental researchers should move beyond reliance on static summary score, while using temporally rich data to explore how parenting effects unfold within families.

The present study offers a significant theoretical extension to the attachment theory by revealing significant effects of daily parenting behaviors on adolescent daily internalizing symptoms via adolescent daily self-compassion. While classical attachment theory posits that early interactions with caregivers lead to the formation of stable internal working models that guide future interpersonal and intrapersonal relationship (Mikulincer & Shaver, 2007), findings of the present study demonstrate that daily parenting behaviors may have short-term effects on daily alterations of adolescents’ internal working models that provide affective-cognitive templates for adolescents to relate to themselves in everyday life. Specifically, adolescents perceiving daily supportive parenting behaviors may concurrently form more positive internal working models of themselves as worthy of care and self-caregiving mental representations, leading to enhanced daily self-warmth that protects against internalizing symptoms on that day. Conversely, adolescents experiencing daily negative controlling parenting behaviors may contemporaneously establish negative internal working models of themselves as undeserving of compassion and internalize negative and harsh treatment from their parents. This renders adolescents to respond to themselves in a similar manner with increased self-criticism and coldness on that day, which in turn amplifies their daily internalizing symptoms.

The differences between concurrent and lagged mediation effects demonstrate that positive effects of supportive parenting behaviors and detrimental effects of negative controlling parenting behaviors are temporary that are unable to last until the following day. The next-day parenting effects were distinct in both valence and significance, indicating that parenting effects might unfold differently over time. These divergent findings suggest the importance of taking multiple “snapshots” with various time intervals to investigate how parenting processes unfold in daily life and the duration of parenting effects. The unexpected concurrent and lagged mediation results illustrate that daily parenting effects on adolescent outcomes should be understood within a broader cultural context, as culture can influence both adolescents’ interpretations of certain parenting behaviors and the functions and meanings of self-compassion (e.g., the positive connotations of self-coldness in China).

Findings can provide crucial practical implications. Daily fluctuations captured in this study is particularly salient, because compared to stable “trait” effects, these daily dynamics are more amenable to change. Examining these dynamic family processes and the underlying mechanism can help understand how and why parenting exert effects, and identify specific, modifiable factors that drive changes in adolescent outcomes. First and foremost, daily parenting behaviors should be enhanced due to their immediate effects on adolescent self-compassion and mental health. Parents should be encouraged to exhibit greater warmth, care and support toward adolescents in everyday life, while avoiding manipulating their adolescents’ behaviors through intrusive psychological control or autocratic control by asserting their authority. It might be particularly relevant and crucial for fathers to reduce strictness as the deleterious effect of paternal strictness was consistently identified across informants. Since significant within-family level mediation effects were primarily found using adolescent-reported parenting behaviors, reframing adolescents’ perceptions of daily parenting may be as important as changing parents’ actual behaviors (Van Lissa et al., 2019). Second, the significant mediation role of daily self-compassion demonstrates it as another important target for alleviating adolescent internalizing symptoms. Since positive and negative facets of self-compassion are distinct constructs, it is essential to simultaneously cultivate adolescent self-warmth while reducing self-coldness in their daily life. Tackling self-coldness might be particularly essential for Chinese adolescents, as they tend to endorse it as a strategy for personal growth at the cost of their mental well-being.

### Limitations and Future Directions

This study addressed key theoretical and methodological limitations of prior research by investigating the mediating role of self-compassion between parenting behaviors and adolescent internalizing symptoms at the daily level. Notwithstanding these contributions, several limitations should be noted. First, although the SSCS-S utilized for assessing daily self-compassion is a validated state measure (Neff et al., 2021), it only contains six items, each representing one component of self-compassion (e.g., self-kindness). This limited number of items may reduce the sensitivity to subtle differences in compassionate or uncompassionate self-relating patterns. Second, the reliabilities of several within-family level parenting measures (e.g., adolescent-reported maternal warmth; *ω* = 0.57) were low (*ω*_*s*_ < 0.60) based on the criteria for trait measures, which may result in an underestimation of the observed effect sizes. However, scholars have argued that more relaxed standards should be applied to within-person reliabilities and *ω* values between 0.41 to 0.60 are deemed fair at the within-person level (Bi et al., 2024; Nezlek, 2017). Third, the adolescent sample of the present study was in the stage of early adolescence. While this could provide insights into daily mediation processes of self-compassion during this specific developmental period, it remains unclear whether these processes remain consistent throughout adolescence. Future studies should replicate these findings in samples from middle and late adolescence. Fourth, while the fixed slope random intercept model utilized in this study could capture average within-family experiences, potential heterogeneity of effects across families might exist, suggesting the need to investigate potential between-family level moderators.

## Conclusion

Attachment theory and empirical evidence support the mediation role of adolescent self-compassion in the relationship between parenting behaviors and adolescent internalizing symptoms at the between-family level. However, as parenting behaviors and adolescent outcomes can fluctuate on a daily basis, how this mediation process operate within families in daily life remains unknown. This daily diary study addressed this gap by examining daily adolescent self-compassion as a within-family mediator in Chinese families. Notwithstanding a few unexpected findings, concurrent indirect results generally show that daily supportive parenting behaviors were associated with lower levels of adolescent internalizing symptoms through increased daily self-warmth, while daily negative controlling behaviors contributed to elevated internalizing symptoms via higher daily self-coldness. The lagged indirect effects yielded distinct results: supportive parenting was prospectively linked to increased internalizing symptoms two days later through higher next-day self-coldness. Most significant results were based on adolescent-perceived rather than parent-reported parenting behaviors. These findings highlight the dynamic nature of parenting processes and show that daily parenting behaviors predict adolescent internalizing symptoms by shaping how adolescents respond to and relate to themselves each day.

## Supplementary information

Supplementary Materials

## Declarations

### Data Sharing Declaration

The data for the current study are not publicly available but are available from the corresponding author on reasonable request.
