# Supplementary material for: Unraveling Daily Linkages between Parenting Behaviors and Adolescent Internalizing Symptoms in Chinese Families: The Mediating Role of Adolescent Self-Compassion
Source: J Youth Adolesc. 2025 Dec 6;55(4):1051–63. doi: 10.1007/s10964-025-02300-x (PMC13076425; doi:10.1007/s10964-025-02300-x)
Supplement: Supplementary file 1 — Supplementary Materials [file 10964_2025_2300_MOESM1_ESM.docx]

**Supplementary Material**

**Unraveling Daily Linkages between Parenting Behaviors and Adolescent Internalizing Symptoms in Chinese Families: The Mediating Role of Adolescent Self-Compassion**

**Table S1**

*Socio-Demographics of Mothers and Fathers*

| *M* (*SD*) or *N* (%) | | *M* (*SD*) or *N* (%) | |
| --- | --- | --- | --- |
| Mothers | | Fathers | |
| Age | 39.48 (3.65) | Age | 42.04 (4.39) |
| Educational level |  | Educational level |  |
| Primary school or below | 1 (1%) | Primary school or below | 0 (0%) |
| Middle school | 5 (5%) | Middle school | 7 (8.23%) |
| Technical secondary school | 9 (9%) | Technical secondary school | 7 (8.23%) |
| High school | 10 (10%) | High school | 8 (9.41%) |
| Associate’ degree | 28 (28%) | Associate’s degree | 21 (24.71%) |
| Bachelor’s degree | 43 (43%) | Bachelor’s degree | 38 (44.71%) |
| Master’s degree | 4 (4%) | Master’s degree | 4 (4.71%) |
| Doctoral degree | 0 (0%) | Doctoral degree | 0 (0%) |
| Annual income (RMB) |  | Annual income (RMB) |  |
| 0-49999 | 22 (22%) | 0-49999 | 6 (7.06%) |
| 50000-99999 | 30 (30%) | 50000-99999 | 14 (16.47%) |
| 100000-149999 | 19 (19%) | 100000-149999 | 24 (28.24%) |
| 150000-199999 | 18 (18%) | 150000-199999 | 19 (22.35%) |
| 200000-249999 | 5 (5%) | 200000-249999 | 8 (9.41%) |
| 250000+ | 6 (6%) | 250000+ | 14 (16.47%) |

*Note.* Socio-demographic categories did not add up to the total number of participants due to missing values.

**Table S2**

*Descriptives, ICCs and Correlations*

|  | Descriptives | | | Correlations | | | | | | | | | | | | | | | | | | | |
| --- | --- | --- | --- | --- | --- | --- | --- | --- | --- | --- | --- | --- | --- | --- | --- | --- | --- | --- | --- | --- | --- | --- | --- |
|  | *M* | *SD* | ICC | 1 | 2 | 3 | 4 | 5 | 6 | 7 | 8 | 9 | 10 | 11 | 12 | 13 | 14 | 15 | 16 | 17 | 18 | 19 | 20 |
| **Child-reported parenting** |  |  |  |  |  |  |  |  |  |  |  |  |  |  |  |  |  |  |  |  |  |  |  |
| 1.Maternal warmth | 7.72 | 2.93 | .55 |  | **.84** | **-.31** | -.08 | **.74** | **.71** | **-.24** | -.14 | **.44** | **.34** | **-.28** | -.07 | .21 | .13 | -.04 | .02 | **.65** | .001 | -.18 | -.13 |
| 2.Maternal autonomy support | 7.56 | 3.23 | .63 | **.59** |  | **-.29** | -.10 | **.67** | **.84** | -.18 | -.12 | **.42** | **.36** | **-.25** | -.12 | **.29** | .21 | -.08 | -.06 | **.61** | .11 | -.10 | -.07 |
| 3.Maternal psychological control | 1.91 | 1.75 | .47 | **-.11** | **-.11** |  | **.39** | **-.27** | -.19 | **.80** | **.47** | **-.31** | **-.25** | **.41** | .22 | -.13 | -.19 | **.46** | **.23** | -.05 | **.41** | **.49** | **.57** |
| 4.Maternal strictness | 3.08 | 2.99 | .55 | **-.11** | **-.09** | **.42** |  | -.10 | -.07 | **.31** | **.73** | -.14 | -.16 | **.24** | **.34** | -.02 | -.04 | **.26** | **.38** | .12 | **.29** | .16 | .11 |
| 5.Paternal warmth | 7.74 | 2.98 | .62 | **.39** | **.38** | **.06** | .02 |  | **.85** | **-.35** | -.15 | **.32** | **.35** | -.19 | .02 | **.42** | **.41** | -.10 | .02 | **.63** | .04 | **-.29** | **-.22** |
| 6.Paternal autonomy support | 7.25 | 3.38 | .70 | **.29** | **.46** | **.06** | .03 | **.72** |  | **-.23** | -.11 | **.34** | **.37** | -.21 | -.07 | **.38** | **.34** | -.14 | -.02 | **.64** | .09 | -.22 | -.14 |
| 7.Paternal psychological control | 1.87 | 1.90 | .59 | **.08** | .04 | **.34** | **.17** | **-.11** | **-.10** |  | **.58** | **-.30** | **-.28** | **.43** | .22 | **-.26** | **-.28** | **.51** | .23 | -.07 | **.41** | **.58** | **.51** |
| 8.Paternal strictness | 2.85 | 3.02 | .62 | .04 | .04 | **.19** | **.25** | **-.15** | **-.13** | **.55** |  | -.15 | -.10 | .19 | **.25** | -.05 | .01 | **.34** | **.48** | .12 | **.32** | **.27** | **.23** |
| **Parent-reported parenting** |  |  |  |  |  |  |  |  |  |  |  |  |  |  |  |  |  |  |  |  |  |  |  |
| 9.Maternal warmth | 8.17 | 2.43 | .71 | -.01 | .03 | .03 | **-.06** | .01 | .02 | .03 | .03 |  | **.88** | **-.42** | .02 | **.44** | **.38** | -.18 | .07 | **.36** | -.12 | -.20 | -.18 |
| 10.Maternal autonomy support | 8.34 | 2.23 | .66 | .03 | .04 | .04 | .001 | -.004 | .05 | **.07** | **.07** | **.53** |  | **-.36** | .02 | **.45** | **.44** | -.20 | .02 | **.35** | -.14 | **-.24** | -.19 |
| 11.Maternal psychological control | 1.90 | 1.43 | .71 | .05 | **.06** | .04 | **.05** | .03 | .06 | .05 | .004 | **-.07** | -.02 |  | **.40** | -.14 | -.11 | **.31** | .06 | -.14 | **.22** | **.28** | **.22** |
| 12.Maternal strictness | 4.49 | 3.23 | .60 | .01 | **-.05** | **.06** | **.10** | .01 | -.02 | .05 | .04 | .02 | **.09** | **.16** |  | .02 | -.01 | .19 | **.37** | .03 | .14 | .08 | .07 |
| 13.Paternal warmth | 7.63 | 2.55 | .71 | **.06** | .06 | -.04 | -.02 | **.09** | .06 | -.02 | .02 | **.15** | **.11** | **-.07** | .03 |  | **.83** | **-.26** | .07 | **.28** | .09 | -.09 | -.07 |
| 14.Paternal autonomy support | 7.73 | 2.60 | .73 | .04 | .05 | -.03 | -.02 | .01 | .04 | .06 | .05 | **.11** | **.09** | -.004 | .02 | **.64** |  | **-.27** | .09 | **.22** | .05 | -.17 | -.09 |
| 15.Paternal psychological control | 2.04 | 1.57 | .73 | .05 | .02 | .04 | .001 | .03 | **.07** | .02 | -.004 | -.02 | -.02 | **.10** | .04 | .01 | -.03 |  | **.44** | .10 | **.41** | **.39** | **.42** |
| 16.Paternal strictness | 4.43 | 3.05 | .69 | .01 | .01 | .05 | .01 | .03 | .04 | .05 | **.07** | -.05 | .03 | .001 | **.10** | **.17** | **.18** | **.22** |  | -.03 | .14 | .09 | .11 |
| **Child-reported self-compassion and outcomes** |  |  |  |  |  |  |  |  |  |  |  |  |  |  |  |  |  |  |  |  |  |  |  |
| 17.Self-warmth | 3.51 | 1.27 | .60 | **.15** | **.20** | **.08** | **.05** | **.20** | **.22** | .004 | .01 | .00 | .03 | .01 | .04 | .05 | .01 | .03 | -.01 |  | **.30** | -.11 | -.02 |
| 18.Self-coldness | 2.10 | 1.20 | .59 | .03 | **.07** | **.13** | **.09** | .04 | **.05** | **.17** | **.09** | -.02 | .01 | -.01 | .03 | .05 | .03 | .05 | **.08** | **.24** |  | **.63** | **.58** |
| 19.Depression symptoms | 2.40 | 2.60 | .50 | -.01 | -.03 | **.23** | **.13** | **-.06** | **-.05** | **.22** | **.15** | **-.07** | .00 | .03 | .04 | **-.07** | -.02 | .05 | .04 | -.03 | **.28** |  | **.85** |
| 20.Anxiety symptoms | 2.58 | 2.67 | .48 | -.01 | -.02 | **.23** | **.14** | **-.06** | -.03 | **.18** | **.12** | **-.05** | .01 | .02 | **.08** | -.04 | -.02 | .04 | .04 | .01 | **.26** | **.63** |  |

*Note.* ICC = intraclass correlation coefficient. Correlation coefficients are standardized. Within-family level correlations are below the diagonal; between-family level correlations are above the diagonal. The boldface coefficients were significant because their 95% credible intervals did not contain zero.

**Table S3**

*Concurrent Within-Level Mediation Results for Adolescent-Reported Parenting with Adolescent Anxiety Symptom as the Outcome*

|  | Path a1 (parenting 🡪 SW) | | Path a2 (parenting 🡪 SC) | | | Path b1 (SW 🡪 Anx) | | | Path b2 (SC 🡪 Anx) | | | Path c’ (parenting 🡪 Anx) | | Indirect effect 1 (mediator: SW) | | Indirect effect 2 (mediator: SC) | |
| --- | --- | --- | --- | --- | --- | --- | --- | --- | --- | --- | --- | --- | --- | --- | --- | --- | --- |
| Model | β | 95% CI | β | 95% CI | | β | | 95% CI | β | 95% CI | | β | 95% CI | *B* | 95% CI | *B* | 95% CI |
| Maternal parenting |  |  |  |  | |  | |  |  |  | |  |  |  |  |  |  |
| Warmth 🡪 SW and SC 🡪 Anx | **.14** | **[.09, .18]** | .04 | [.00, .08] | | **-.06** | | **[-.10, -.02]** | **.26** | **[.22, .30]** | | -.02 | [-.07, .02] | **-.007** | **[-.01, -.002]** | .01 | [.00, .02] |
| Autonomy support 🡪 SW and SC 🡪 Anx | **.17** | **[.13, .22]** | **.07** | **[.02, .11]** | | **-.06** | | **[-.10, -.01]** | **.26** | **[.22, .30]** | | -.03 | [-.07, .02] | **-.01** | **[-.02, -.002]** | **.02** | **[.01, .03]** |
| Psychological control 🡪 SW and SC 🡪 Anx | **.07** | **[.02, .11]** | **.13** | **[.09, .17]** | | **-.07** | | **[-.11, -.03]** | **.23** | **[.19, .27]** | | **.19** | **[.15, .23]** | **-.006** | **[-.01, -.002]** | **.04** | **[.03, .06]** |
| Strictness 🡪 SW and SC 🡪 Anx | .04 | [-.01, .08] | **.09** | **[.05, .13]** | | **-.06** | | **[-.10, -.02]** | **.25** | **[.21, .29]** | | **.11** | **[.07, .16]** | -.002 | [-.01, .00] | **.02** | **[.01, .03]** |
| Paternal parenting |  |  |  |  | |  | |  |  |  | |  |  |  |  |  |  |
| Warmth 🡪 SW and SC 🡪 Anx | **.17** | **[.13, .22]** | **.05** | **[.004, .10]** | | **-.05** | | **[-.09, -.01]** | **.26** | **[.22, .30]** | | **-.07** | **[-.12, -.02]** | **-.01** | **[-.02, -.001]** | **.01** | **[.001, .03]** |
| Autonomy support 🡪 SW and SC 🡪 Anx | **.19** | **[.15, .24]** | **.05** | **[.001, .09]** | | **-.05** | | **[-.09, -.01]** | **.26** | **[.22, .30]** | | **-.05** | **[-.10, -.001]** | **-.01** | **[-.02, -.001]** | .01 | [.00, .03] |
| Psychological control 🡪 SW and SC 🡪 Anx | -.01 | [-.05, .04] | **.16** | **[.11, .20]** | **-.06** | | **[-.10, -.02]** | | **.23** | | **[.19, .28]** | **.13** | **[.08, .17]** | .00 | [-.004, .01] | **.05** | **[.04, .07]** |
| Strictness 🡪 SW and SC 🡪 Anx | .01 | [-.03, .06] | **.09** | **[.05, .14]** | **-.06** | | **[-.10, -.02]** | | **.25** | | **[.21, .29]** | **.08** | **[.04, .13]** | -.001 | [-.004, .002] | **.02** | **[.01, .04]** |

*Note.* SW = self-warmth; SC = self-coldness; Anx = anxiety. β for each path was standardized coefficient; *B* for each indirect effect was unstandardized coefficient. The boldface coefficients were significant according to 95% credible intervals.

**Table S4**

*Lagged Within-Level Mediation Results for Adolescent-Reported Parenting with Adolescent Anxiety Symptom as the Outcome*

|  | Path a1 (parenting 🡪 SW) | | Path a2 (parenting 🡪 SC) | | Path b1 (SW 🡪 Anx) | | Path b2 (SC 🡪 Anx) | | Path c’ (parenting 🡪 Anx) | | Indirect effect 1 (mediator: SW) | | Indirect effect 2 (mediator: SC) | |
| --- | --- | --- | --- | --- | --- | --- | --- | --- | --- | --- | --- | --- | --- | --- |
| Model | β | 95% CI | β | 95% CI | β | 95% CI | β | 95% CI | β | 95% CI | *B* | 95% CI | *B* | 95% CI |
| Maternal parenting |  |  |  |  |  |  |  |  |  |  |  |  |  |  |
| Warmth (t-2) 🡪 SW and SC (t-1) 🡪 Anx (t) | .03 | [-.02, .08] | **.05** | **[.004, .09]** | -.01 | [-.05, .04] | **.06** | **[.01, .10]** | **.06** | **[.01, .11]** | .00 | [-.003, .001] | .002 | [.00, .01] |
| Autonomy support (t-2) 🡪 SW and SC (t-1) 🡪 Anx (t) | .04 | [-.01, .09] | **.08** | **[.03, .12]** | -.004 | [-.05, .04] | **.06** | **[.01, .10]** | .02 | [-.03, .07] | .00 | [-.002, .002] | **.004** | **[.001, .01]** |
| Psychological control (t-2) 🡪 SW and SC (t-1) 🡪 Anx (t) | .02 | [-.03, .07] | -.002 | [-.05, .05] | -.01 | [-.06, .03] | **.06** | **[.01, .10]** | .01 | [-.04, .06] | .00 | [-.003, .002] | .00 | [-.01, .004] |
| Strictness (t-2) 🡪 SW and SC (t-1) 🡪 Anx (t) | .02 | [-.02, .07] | -.01 | [-.06, .03] | -.01 | [-.06, .03] | **.06** | **[.02, .11]** | -.001 | [-.05, .05] | .00 | [-.002, .001] | -.001 | [-.004, .002] |
| Paternal parenting |  |  |  |  |  |  |  |  |  |  |  |  |  |  |
| Warmth (t-2) 🡪 SW and SC (t-1) 🡪 Anx (t) | .05 | [.00, .10] | **.08** | **[.03, .13]** | -.002 | [-.05, .04] | **.06** | **[.01, .10]** | .02 | [-.03, .07] | .00 | [-.003, .003] | **.004** | **[.001, .01]** |
| Autonomy support (t-2) 🡪 SW and SC (t-1) 🡪 Anx (t) | **.05** | **[.001, .11]** | **.07** | **[.02, .12]** | -.01 | [-.05, .04] | **.05** | **[.01, .10]** | .05 | [-.01, .10] | .00 | [-.003, .002] | .004 | [.00, .01] |
| Psychological control (t-2) 🡪 SW and SC (t-1) 🡪 Anx (t) | .01 | [-.04, .06] | .03 | [-.02, .08] | -.01 | [-.05, .04] | **.06** | **[.01, .10]** | **.05** | **[.002, .10]** | .00 | [-.003, .002] | .002 | [-.002, .01] |
| Strictness (t-2) 🡪 SW and SC (t-1) 🡪 Anx (t) | .00 | [-.05, .05] | .02 | [-.03, .07] | -.01 | [-.05, .04] | **.06** | **[.01, .11]** | .03 | [-.02, .08] | .00 | [-.001, .001] | .001 | [-.002, .01] |

*Note.* SW = self-warmth; SC = self-coldness; Anx = anxiety. β for each path was standardized coefficient; *B* for each indirect effect was unstandardized coefficient. The boldface coefficients were significant according to 95% credible intervals.

**Table S5**

*Concurrent Within-Level Mediation Results for Parent-Reported Parenting with Adolescent Depression Symptom as the Outcome*

|  | Path a1 (parenting 🡪 SW) | | Path a2 (parenting 🡪 SC) | | | Path b1 (SW 🡪 Dep) | | | Path b2 (SC 🡪 Dep) | | | Path c’ (parenting 🡪 Dep) | | Indirect effect 1 (mediator: SW) | | Indirect effect 2 (mediator: SC) | |
| --- | --- | --- | --- | --- | --- | --- | --- | --- | --- | --- | --- | --- | --- | --- | --- | --- | --- |
| Model | β | 95% CI | β | 95% CI | | β | | 95% CI | β | 95% CI | | β | 95% CI | *B* | 95% CI | *B* | 95% CI |
| Maternal parenting |  |  |  |  | |  | |  |  |  | |  |  |  |  |  |  |
| Warmth 🡪 SW and SC 🡪 Dep | -.001 | [-.05, .04] | -.02 | [-.06, .03] | | **-.11** | | **[-.15, -.07]** | **.28** | **[.24, .32]** | | **-.06** | **[-.10, -.02]** | .00 | [-.01, .01] | -.01 | [-.03, .01] |
| Autonomy support 🡪 SW and SC 🡪 Dep | .03 | [-.02, .07] | .02 | [-.03, .07] | | **-.11** | | **[-.15, -.07]** | **.28** | **[.25, .32]** | | .01 | [-.04, .05] | -.004 | [-.01, .003] | .01 | [-.01, .03] |
| Psychological control 🡪 SW and SC 🡪 Dep | .003 | [-.04, .05] | -.01 | [-.05, .04] | | **-.11** | | **[-.15, -.07]** | **.29** | **[.25, .32]** | | .04 | [-.01, .08] | -.001 | [-.01, .01] | -.003 | [-.03, .03] |
| Strictness 🡪 SW and SC 🡪 Dep | .02 | [-.02, .07] | .02 | [-.03, .06] | | **-.11** | | **[-.15, -.07]** | **.28** | **[.25, .32]** | | .02 | [-.02, .06] | -.002 | [-.01, .002] | .01 | [-.01, .02] |
| Paternal parenting |  |  |  |  | |  | |  |  |  | |  |  |  |  |  |  |
| Warmth 🡪 SW and SC 🡪 Dep | .05 | [-.001, .10] | .04 | [-.01, .09] | | **-.10** | | **[-.14, -.06]** | **.29** | **[.25, .33]** | | **-.06** | **[-.11, -.01]** | -.006 | [-.01, .00] | .01 | [-.004, .03] |
| Autonomy support 🡪 SW and SC 🡪 Dep | .01 | [-.04, .06] | .02 | [-.03, .07] | | **-.11** | | **[-.14, -.07]** | **.28** | **[.25, .32]** | | -.03 | [-.07, .02] | -.001 | [-.01, .01] | .01 | [-.01, .03] |
| Psychological control 🡪 SW and SC 🡪 Dep | .03 | [-.02, .09] | .04 | [-.01, .10] | **-.11** | | **[-.15, -.07]** | | **.28** | | **[.24, .32]** | .03 | [-.02, .08] | -.01 | [-.02, .004] | .03 | [-.01, .06] |
| Strictness 🡪 SW and SC 🡪 Dep | -.001 | [-.05, .05] | **.07** | **[.02, .13]** | **-.11** | | **[-.14, -.07]** | | **.29** | | **[.25, .32]** | -.01 | [-.06, .04] | .00 | [-.01, .01] | **.02** | **[.01, .04]** |

*Note.* SW = self-warmth; SC = self-coldness; Dep = depression. β for each path was standardized coefficient; *B* for each indirect effect was unstandardized coefficient. The boldface coefficients were significant according to 95% credible intervals.

**Table S6**

*Lagged Within-Level Mediation Results for Parent-Reported Parenting with Adolescent Depression Symptom as the Outcome*

|  | Path a1 (parenting 🡪 SW) | | Path a2 (parenting 🡪 SC) | | Path b1 (SW 🡪 Dep) | | Path b2 (SC 🡪 Dep) | | Path c’ (parenting 🡪 Dep) | | Indirect effect 1 (mediator: SW) | | Indirect effect 2 (mediator: SC) | |
| --- | --- | --- | --- | --- | --- | --- | --- | --- | --- | --- | --- | --- | --- | --- |
| Model | β | 95% CI | β | 95% CI | β | 95% CI | β | 95% CI | β | 95% CI | *B* | 95% CI | *B* | 95% CI |
| Maternal parenting |  |  |  |  |  |  |  |  |  |  |  |  |  |  |
| Warmth (t-2) 🡪 SW and SC (t-1) 🡪 Dep (t) | .01 | [-.03, .06] | -.03 | [-.07, .02] | .02 | [-.03, .06] | **.07** | **[.02, .11]** | **-.06** | **[-.11, -.02]** | .00 | [-.001, .003] | -.002 | [-.01, .002] |
| Autonomy support (t-2) 🡪 SW and SC (t-1) 🡪 Dep (t) | .01 | [-.04, .06] | -.02 | [-.06, .03] | .02 | [-.03, .06] | **.07** | **[.02, .12]** | .02 | [-.02, .07] | .00 | [-.002, .003] | -.002 | [-.01, .003] |
| Psychological control (t-2) 🡪 SW and SC (t-1) 🡪 Dep (t) | .01 | [-.04, .06] | .01 | [-.04, .05] | .02 | [-.03, .06] | **.07** | **[.02, .12]** | .04 | [-.01, .08] | .00 | [-.002, .004] | .001 | [-.01, .01] |
| Strictness (t-2) 🡪 SW and SC (t-1) 🡪 Dep (t) | -.03 | [-.07, .02] | -.01 | [-.05, .04] | .02 | [-.02, .06] | **.07** | **[.02, .12]** | **.06** | **[.01, .10]** | .00 | [-.003, .001] | .00 | [-.004, .003] |
| Paternal parenting |  |  |  |  |  |  |  |  |  |  |  |  |  |  |
| Warmth (t-2) 🡪 SW and SC (t-1) 🡪 Dep (t) | .03 | [-.02, .08] | -.003 | [-.05, .05] | .02 | [-.03, .06] | **.07** | **[.02, .12]** | .01 | [-.04, .05] | .00 | [-.001, .004] | .00 | [-.01, .01] |
| Autonomy support (t-2) 🡪 SW and SC (t-1) 🡪 Dep (t) | -.01 | [-.06, .05] | -.01 | [-.06, .04] | .02 | [-.03, .06] | **.07** | **[.02, .12]** | -.01 | [-.06, .04] | .00 | [-.003, .002] | -.001 | [-.01, .004] |
| Psychological control (t-2) 🡪 SW and SC (t-1) 🡪 Dep (t) | -.002 | [-.06, .05] | .02 | [-.03, .07] | .02 | [-.03, .06] | **.07** | **[.02, .12]** | .01 | [-.04, .06] | .00 | [-.004, .004] | .003 | [-.01, .01] |
| Strictness (t-2) 🡪 SW and SC (t-1) 🡪 Dep (t) | -.02 | [-.08, .03] | .05 | [-.01, .10] | .02 | [-.03, .06] | **.07** | **[.02, .12]** | .02 | [-.03, .07] | .00 | [-.003, .001] | .003 | [.00, .01] |

*Note.* SW = self-warmth; SC = self-coldness; Dep = depression. β for each path was standardized coefficient; *B* for each indirect effect was unstandardized coefficient. The boldface coefficients were significant according to 95% credible intervals.

**Table S7**

*Concurrent Within-Level Mediation Results for Parent-Reported Parenting with Adolescent Anxiety Symptom as Outcome*

|  | Path a1 (parenting 🡪 SW) | | Path a2 (parenting 🡪 SC) | | | Path b1 (SW 🡪 Anx) | | | Path b2 (SC 🡪 Anx) | | | Path c’ (parenting 🡪 Anx) | | Indirect effect 1 (mediator: SW) | | Indirect effect 2 (mediator: SC) | |
| --- | --- | --- | --- | --- | --- | --- | --- | --- | --- | --- | --- | --- | --- | --- | --- | --- | --- |
| Model | β | 95% CI | β | 95% CI | | β | | 95% CI | β | 95% CI | | β | 95% CI | *B* | 95% CI | *B* | 95% CI |
| Maternal parenting |  |  |  |  | |  | |  |  |  | |  |  |  |  |  |  |
| Warmth 🡪 SW and SC 🡪 Anx | -.001 | [-.05, .04] | -.02 | [-.07, .03] | | **-.06** | | **[-.10, -.02]** | **.26** | **[.22, .29]** | | **-.05** | **[-.09, -.004]** | .00 | [-.004, .01] | -.01 | [-.03, .01] |
| Autonomy support 🡪 SW and SC 🡪 Anx | .03 | [-.02, .07] | .02 | [-.03, .06] | | **-.06** | | **[-.10, -.02]** | **.26** | **[.22, .30]** | | .00 | [-.05, .05] | -.002 | [-.01, .002] | .01 | [-.01, .02] |
| Psychological control 🡪 SW and SC 🡪 Anx | .003 | [-.04, .05] | -.01 | [-.05, .04] | | **-.06** | | **[-.10, -.02]** | **.26** | **[.22, .30]** | | .04 | [-.01, .09] | .00 | [-.01, .01] | -.003 | [-.03, .02] |
| Strictness 🡪 SW and SC 🡪 Anx | .02 | [-.02, .07] | .02 | [-.02, .06] | | **-.06** | | **[-.10, -.02]** | **.26** | **[.22, .30]** | | **.07** | **[.02, .11]** | -.001 | [-.01, .001] | .01 | [-.01, .02] |
| Paternal parenting |  |  |  |  | |  | |  |  |  | |  |  |  |  |  |  |
| Warmth 🡪 SW and SC 🡪 Anx | .05 | [.00, .10] | .04 | [-.01, .09] | | **-.06** | | **[-.10, -.02]** | **.26** | **[.22, .30]** | | -.04 | [-.08, .01] | -.004 | [-.01, .00] | .01 | [-.004, .03] |
| Autonomy support 🡪 SW and SC 🡪 Anx | .01 | [-.04, .06] | .02 | [-.03, .07] | | **-.06** | | **[-.10, -.02]** | **.26** | **[.22, .30]** | | -.03 | [-.08, .02] | -.001 | [-.01, .004] | .01 | [-.01, .03] |
| Psychological control 🡪 SW and SC 🡪 Anx | .03 | [-.02, .08] | .04 | [-.01, .10] | **-.06** | | **[-.10, -.02]** | | **.26** | | **[.22, .30]** | .02 | [-.04, .07] | -.004 | [-.01, .003] | .02 | [-.01, .06] |
| Strictness 🡪 SW and SC 🡪 Anx | -.001 | [-.05, .05] | **.08** | **[.02, .13]** | **-.06** | | **[-.10, -.02]** | | **.26** | | **[.22, .90]** | .01 | [-.04, .06] | .00 | [-.004, .004] | **.02** | **[.01, .04]** |

*Note.* SW = self-warmth; SC = self-coldness; Anx = anxiety. β for each path was standardized coefficient; *B* for each indirect effect was unstandardized coefficient. The boldface coefficients were significant according to 95% credible intervals.

**Table S8**

*Lagged Within-Level Mediation Results for Parent-Reported Parenting with Adolescent Anxiety Symptom as Outcome*

|  | Path a1 (parenting 🡪 SW) | | Path a2 (parenting 🡪 SC) | | Path b1 (SW 🡪 Anx) | | Path b2 (SC 🡪 Anx) | | Path c’ (parenting 🡪 Anx) | | Indirect effect 1 (mediator: SW) | | Indirect effect 2 (mediator: SC) | |
| --- | --- | --- | --- | --- | --- | --- | --- | --- | --- | --- | --- | --- | --- | --- |
| Model | β | 95% CI | β | 95% CI | β | 95% CI | β | 95% CI | β | 95% CI | *B* | 95% CI | *B* | 95% CI |
| Maternal parenting |  |  |  |  |  |  |  |  |  |  |  |  |  |  |
| Warmth (t-2) 🡪 SW and SC (t-1) 🡪 Anx (t) | .01 | [-.03, .06] | -.02 | [-.07, .02] | -.01 | [-.05, .04] | **.06** | **[.01, .11]** | -.04 | [-.09, .01] | .00 | [-.002, .002] | -.002 | [-.01, .002] |
| Autonomy support (t-2) 🡪 SW and SC (t-1) 🡪 Anx (t) | .01 | [-.04, .06] | -.02 | [-.06, .04] | -.01 | [-.05, .04] | **.06** | **[.01, .11]** | .00 | [-.05, .05] | .00 | [-.002, .002] | -.001 | [-.01, .003] |
| Psychological control (t-2) 🡪 SW and SC (t-1) 🡪 Anx (t) | .01 | [-.04, .06] | .004 | [-.04, .05] | -.01 | [-.05, .04] | **.06** | **[.01, .11]** | .03 | [-.02, .08] | .00 | [-.003, .003] | .00 | [-.01, .01] |
| Strictness (t-2) 🡪 SW and SC (t-1) 🡪 Anx (t) | -.03 | [-.08, .02] | -.02 | [-.07, .03] | -.01 | [-.05, .04] | **.06** | **[.01, .11]** | **.06** | **[.01, .10]** | .00 | [-.002, .002] | -.001 | [-.01, .001] |
| Paternal parenting |  |  |  |  |  |  |  |  |  |  |  |  |  |  |
| Warmth (t-2) 🡪 SW and SC (t-1) 🡪 Anx (t) | .03 | [-.02, .08] | -.02 | [-.06, .04] | -.01 | [-.05, .04] | **.06** | **[.01, .11]** | -.001 | [-.05, .05] | .00 | [-.003, .002] | -.001 | [-.01, .003] |
| Autonomy support (t-2) 🡪 SW and SC (t-1) 🡪 Anx (t) | -.003 | [-.06, .05] | -.02 | [-.07, .03] | -.01 | [-.05, .04] | **.06** | **[.01, .11]** | -.02 | [-.08, .03] | .00 | [-.002, .002] | -.001 | [-.01, .003] |
| Psychological control (t-2) 🡪 SW and SC (t-1) 🡪 Anx (t) | -.001 | [-.06, .05] | .02 | [-.03, .07] | -.01 | [-.05, .04] | **.06** | **[.01, .11]** | .05 | [-.01, .10] | .00 | [-.003, .003] | .002 | [-.005, .01] |
| Strictness (t-2) 🡪 SW and SC (t-1) 🡪 Anx (t) | -.02 | [-.08, .03] | .04 | [-.02, .09] | -.01 | [-.05, .04] | **.06** | **[.01, .11]** | .02 | [-.04, .07] | .00 | [-.002, .002] | .002 | [-.001, .01] |

*Note.* SW = self-warmth; SC = self-coldness; Anx = anxiety. β for each path was standardized coefficient; *B* for each indirect effect was unstandardized coefficient. The boldface coefficients were significant according to 95% credible intervals.
